# High-Dielectric PVP@PANI/PDMS Composites Fabricated via an Electric Field-Assisted Approach

**DOI:** 10.3390/polym14204381

**Published:** 2022-10-17

**Authors:** Huaixiao Wei, Yuan Yuan, Tianli Ren, Lijuan Zhou, Xueqing Liu, Haroon A. M. Saeed, Pingliang Jin, Yuwei Chen

**Affiliations:** 1 Key Laboratory of Rubber-Plastics, Ministry of Education/Shandong Provincial Key Laboratory of Rubber-Plastics, Qingdao University of Science & Technology, Qingdao 266042, China; 2 Mississippi Polymer Institute, The University of Southern Mississippi, Hattiesburg, MS 39406, USA; 3 Key Laboratory of Optoelectronic Chemical Materials and Devices, Ministry of Education and Flexible Display Materials and Technology Co-Innovation Centre of Hubei Province, Jianghan University, Wuhan 430056, China; 4 The Centre of Fibres, Papers, and Recycling, Faculty of Industries Engineering and Technology, University of Gezira, Wad Medani P.O. Box 20, Sudan; 5 Shanghaitex Architectural Design Research Institute Limited Company, Shanghai 200060, China

**Keywords:** electric fields, alignment, high dielectric, PANI, PDMS

## Abstract

Polymer-based composite films with multiple properties, such as low dielectric loss tangent, high dielectric constant, and low cost are promising materials in the area of electronics and electric industries. In this study, flexible dielectric films were fabricated via an electric field-assisted method. Polyaniline (PANI) was modified by polyvinylpyrrolidone (PVP) to form a core–shell structure to serve as functional particles and silicone rubber polydimethylsiloxane (PDMS) served as the matrix. The dielectric constant of the composites prepared under electric fields was improved by the micro-structures formed by external electric fields. With the addition of 2.5 wt% PVP@PANI, the dielectric constant could be significantly enhanced, up to 23; the dielectric loss tangent is only 1, which is lower than that of the aligned PANI samples. This new processing technology provides important insights for aligning fillers in polymer matrix to form composites with enhanced dielectric properties.

## 1. Introduction

With the development of electronic technology, the preparation of electronic materials with high dielectric property has attracted lots of attention. Compared to traditional ceramic-based dielectric materials, polymer composites have the advantages of easy processing, good flexibility, light weight, and low cost, and have become the mainstream material of the microelectronics industry in recent years [1,2,3,4]. Adding high-dielectric fillers to polymers can significantly increase the dielectric constant of the composites; however, high filler loading leads to higher dielectric loss tangent, which limits the application of composites [5,6,7,8]. Therefore, the preparation of polymer composite materials with high dielectric constant and low dielectric loss tangent is still a challenge [9,10,11,12,13].

Incorporating high-dielectric ceramic particles into the polymer matrix is a classical composite with enhanced dielectric property. In order to restrain the dielectric loss tangent caused by the addition of fillers, a transition layer is usually employed on the filler surface. For instance, dopamine-coated barium titanate particles (DP-BT) were introduced into silicone rubber (SR) to form high-dielectric composites [14]. The composite of SR/DP-BT exhibits a dielectric constant as high as approximately 7.9 at 1 kHz when the filler content is 40 wt%. Gall et al. [15] explored the electrical properties of lead magnesium niobate–lead titanate/silicone elastomer (PMN-PT/PDMS) composites which were prepared by heat curing. The dielectric constant of 30 wt% PMN-PT/PDMS composites increased from 8 to 32 at 10 Hz. The problem with the aforementioned strategy is that the dielectric constants of the composites only effectively increase when the addition of dielectric ceramic fillers in composite materials is very high (up to 40–50 vol%). Excessive addition is likely to cause holes and defects in the composite material, which leads to difficulty in processing and reduction of mechanical strength, and at the same time, the cost is high, which is not conducive to the practical application of these types of materials. Another strategy is to disperse conductive particles into a polymer matrix. Cameron et al. [16] added conductive graphite to polyurethane and observed high dielectric constant (4400) at a volume fraction of 18.76% graphite loading. Chen et al. [17,18,19,20,21] also prepared a series of modified graphite/polymer matrix composites with excellent dielectric properties with high dielectric constant and low dielectric loss tangent. The mechanism of these conductive filler/polymer matrix composites is that the dielectric constant of polymer composites can be significantly increased as the loading of conductive fillers increases around the permeation threshold. However, when the conductive particles exceed the permeation threshold in the matrix, which means the conductive fillers are interconnected to form a continuous conductive path or network, the polymer material undergoes an insulator–conductor transition and the dielectric loss tangent increases rapidly, which causes considerable difficulties and challenges in obtaining reproducible and stable products for practical applications [22,23].

Conductor–insulator core–shell particles have been used as ideal high-k filler particles in polymer composites. Due to the interface polarization, the conductor is used to increase the dielectric constant, while the insulating shell acts as a dielectric interlayer, which can effectively reduce the dielectric loss tangent by blocking electron transfer between adjacent conductors. For instance, Zhang et al. investigated polyaniline-coated calcium copper titanate (CCTO@PANI) core–shell particles as additives within PDMS to prepare CCTO@PANI/PDMS composites. The experimental results showed that the introduction of core–shell structured fillers increased the dielectric constant and suppressed the increasement of dielectric loss tangent [24]. Dang et al. [25] prepared Ag@ TiO_2_ /PVDF nanocomposite films by solvent casting method. The Ag@TiO_2_ core@shell nanoparticles were synthesized via a water-thermal meting route. A certain amount of Ag@TiO_2_ nanoparticles and PVDF were ultrasonically dispersed in the organic solvent DMF. Afterwards, the solution was heated to completely evaporate the solvent, and subsequently molded by hot-pressing. The dielectric constant increases significantly with increasing filler content since the silver forms innumerable tiny micro-capacitors in the matrix. In a study by Silakaew et al., Ag@BaTiO_3_ and RuO_2_@BaTiO_3_ particles were prepared by surface adsorption deposition to promote the dielectric response of polyvinylidene fluoride (PVDF). The dielectric constant of the composites increased with the particle volume fraction, while the loss tangent was effectively suppressed. A higher dielectric constant was obtained, while a low loss tangent was obtained [26,27]. In a study by Plattke et al., a novel dielectric core-satellite BT-gold (Au) nanoparticle (NP) was prepared by a surface chemical reaction method for use as a filler in PVDF to improve the dielectric constant, energy density, and efficiency of composites while reducing dielectric loss [28]. Nevertheless, the high modulus of the insulating shell leads to defects in the composites, which inevitably limits the properties of the composites. Given these shortcomings, all-organic fillers and external field assistance have been developed in recent years. The use of all-organic fillers and external field assistance has the following two advantages: (i) the insulating polymer has a low modulus on the basis of insulating properties, is compatible with the polymer matrix, and is easy to disperse; and (ii) the external field-assisted orientation makes it easier for the composites to form anisotropic micro-structures, which can enhance the dielectric properties and reduce the filler loading of the composites [29].

In this study, high dielectric polymer composites fabricated by a combination of conductive–insulative core–shell structure design and electric field-assisted self-assembly approach. PVP@PANI fillers were prepared by using conductive polyaniline (PANI) as the core and insulative polyvinylpyrrolidone (PVP) as the layer, then assembled into micro-structures to further enhance the dielectric property of the composite. The dielectric constant of the composites prepared under electric fields was improved by the micro-structures formed by external electric fields. With the addition of 2.5 wt% PVP@PANI, the dielectric constant could be significantly enhanced up to 23.

## 2. Experimental Section

### 2.1. Materials

PVP K30 purchased from Sinopharm Chemical Reagent Co., Ltd (Shanghai, China). PANI with an average particle size of around 30 microns was provided by Anhui Kuer Biological Engineering Co., Ltd (Hefei, China). The polydimethylsiloxane (PDMS) base and curing agent (Sylgard 184) were supplied by Dow Corning (Midland, Michigan, USA). The PDMS and curing agent were mixed in a mass ratio of 10:1, and then cured at 90 °C for 1 h. The indium tin oxide (ITO)-coated glass substrate was purchased from Xiang Technology Co., Ltd (Guangzhou, China). It has good transparent conductivity, high light transmittance, and low resistivity in the visible spectral region, so it is used as an electrode for electric field-assisted alignment.

### 2.2. Preparation of PVP@PANI/PDMS Composites

PVP@PANI fillers were prepared by surface physical modification, as reported by Liu et al. [30]. A total of 5 g of PANI was dissolved in 50 mL of ethanol provided by Qingdao Eurasia Chemical Technology Development Co., Ltd (Qingdao, China) and ultrasonicated for 1 h, then 0.25 g of PVP was dissolved in 40 mL of ethanol and ultrasonicated for 1 h. The PVP-ethanol solution was mixed with PANI-ethanol solution. After 10 h of mechanical stirring the combined solution was centrifuged 4 times at 8000 r/min, then vacuum dried at 80 °C for 8 h.

PVP@PANI fillers were dispersed in PDMS matrix by applying a planetary centrifugal mixer (ZYMC-200V, ZYE Science &Technology, Shenzhen, China) to homogenize the mixed solution. A square cell consisting of two ITO coated conductive glass electrodes was separated by a 1 mm spacer. The top electrode was connected to a high-voltage amplifier (AMJ-2B10, Matsusada, Osaka, Japan) with a function generator (HDG2012B, Hantek, Qingdao, China), and the bottom electrode was grounded. The suspension was then poured into the cell and an electric field of 1000 V p-p/cm and 10 Hz was applied. After applying the electric field for 1 min, the PDMS composites were cured on a hot plate at 90 °C for 1 h to freeze the assembled microstructures. An oscilloscope (DSO5072P, Hantek, Qingdao, China) was used to monitor electric field signals throughout the manufacturing process. Control samples were prepared using the same experimental method in the absence of an electric field.

### 2.3. Characterization

A Bruker VERTEX 70 spectrometer (Bruker, Karlsruhe, Germany) was employed to obtain the Fourier transform infrared (FTIR) spectrum of compounds under a scanning range from 400 to 2000 cm^−1^, with a resolution of 4 cm^−1^. The surface shape of the samples was investigated on a transmission electron microscope (TEM, JOEL, JEM-2100, Peabody, MA, USA) at 200 kV. The morphology of the as-prepared composite films was characterized by scanning electron microscopy (SEM, 7500F, JEOL, Peabody, MA, USA). Cross-sectional samples were prepared by liquid nitrogen cryofracturing. The orientation process of the particles under the electric field was observed in situ using an optical microscope (MP41, Mshot, Mingmei Optoelectronic Technology Co., Ltd, Guangzhou, China). The dielectric constant, dielectric loss tangent, and electrical conductivity of the composite elastomers were measured using a broadband dielectric spectrometer (Alpha-A, Novocontrol Technologies GmbH & Co. KG, Montabauer, Germany) in the frequency range of 10^2^ to 10^7^ Hz at room temperature. The dielectric properties and electrical conductivity of the material were measured by broadband dielectric spectrometer with copper electrode. The sample diameter was 25 mm and the thickness was 1 mm.

## 3. Results and Discussion

### 3.1. Morphology of the PVP@PANI Particle

**Figure 1:**
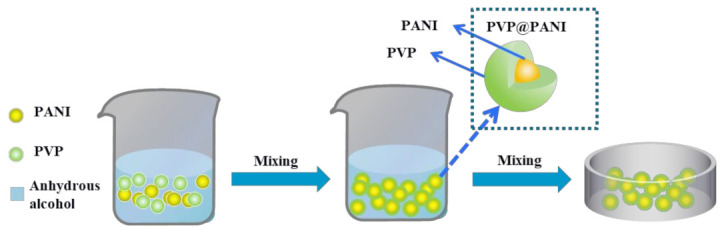
Schematic illustration for the preparation of PVP@PANI.

**Figure 2:**
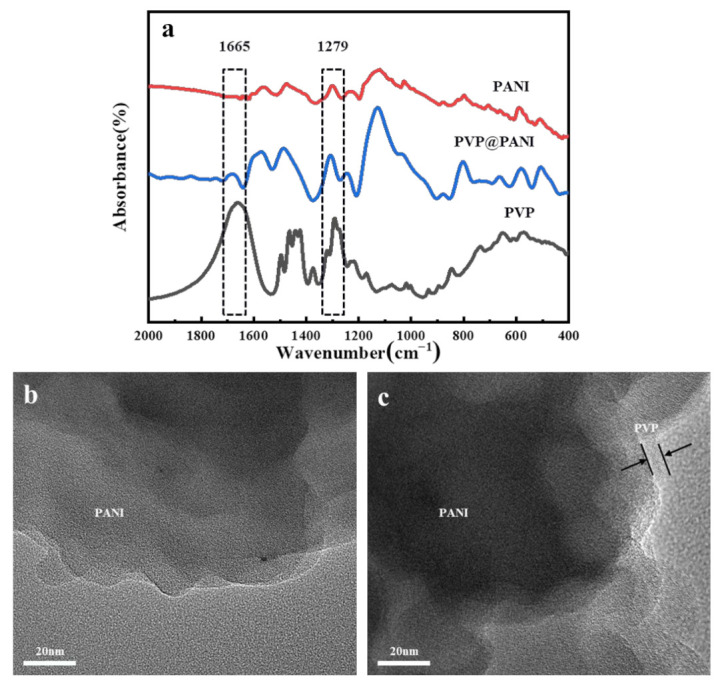
Infrared spectra of PANI, PVP@PANI, and PVP (**a**). TEM images of PANI (**b**) and PVP@PANI (**c**).

As shown in Figure 1, the experimental method of surface physical modification was used to modify the PANI particles with PVP, which is denoted as PVP@PANI. The structure of PVP@PANI particles was analyzed by infrared spectroscopy and the dispersion of PVP@PANI particles in the matrix was investigated using scanning electron microscopy. The infrared spectra of polyaniline, polyvinylpyrrolidone, and polyvinylpyrrolidone-coated polyaniline are shown in Figure 2a. The peak at 1665 cm^−1^ is the band generated by the carbonyl-C=O vibration in PVP, and 1279 cm^−1^ is the stretching vibration peak of the CN bond of PVP [31]. These two peaks exist in the PVP@PANI spectrum at the same time. Figure 2b is the high-resolution transmission image of PANI, and Figure 2c is the high-resolution transmission image of PVP@PANI. Compared with the surface of PANI in Figure 2b, there is a relatively transparent film on the surface of PANI in Figure 2c. Combined with the infrared spectrum, it can be seen that PVP has been successfully coated onto PANI.

**Figure 3:**
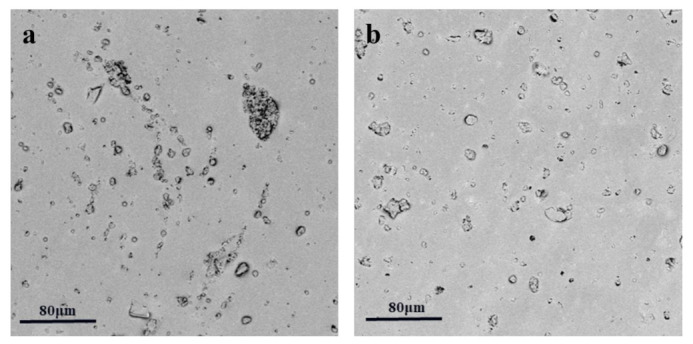
SEM images of dielectric composites PANI/PDMS (**a**), and PVP@PANI/PDMS (**b**).

Figure 3a,b are SEM images of the composites. Figure 3a shows the dispersion phenomenon of unmodified polyaniline in the matrix, with some degree of agglomeration. Figure 3b shows the dispersion phenomenon of the modified polyaniline in the matrix. The example is uniformly dispersed, which verifies that PVP successfully modified the polyaniline from the side.

### 3.2. Micro-Structures of PVP@PANI/PDMS Composites

**Figure 4:**
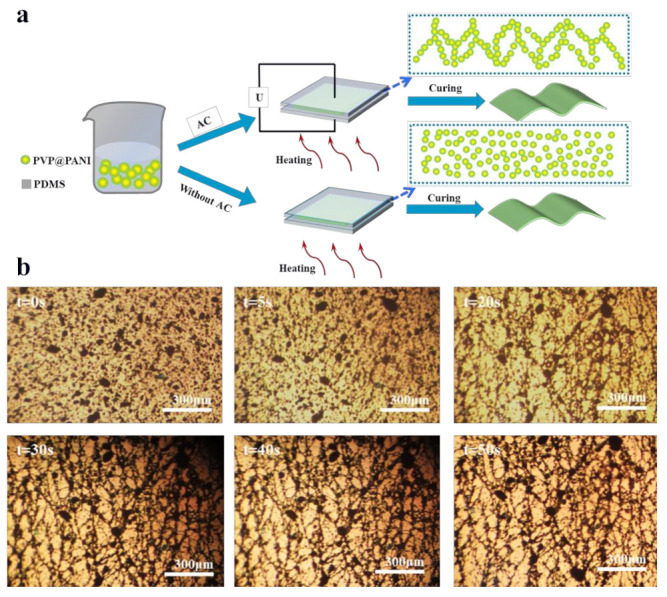
Schematic diagram of the preparation of PVP@PANI/PDMS composites. (**a**) Preparation of PVP@PANI/PDMS composites. (**b**) Micrograph of PVP@PANI particles to form a network structure in a PDMS matrix under electric field strength of 1000 V p-p/mm and frequency of 10 Hz.

Figure 4a illustrates the process of preparing high dielectric constant polymer composites by incorporating PVP@PANI particles into a PDMS matrix. The PVP@PANI particles are polarized and then the network structure is spontaneously assembled within the PDMS matrix. For the control samples prepared without applying an electric field, PVP@PANI particles were randomly dispersed in PDMS matrix. Figure 4b shows micro-network structures of the particles. Since the particles in the assembled composite formed a multi-layer network structure in the matrix, the SEM images showed random dispersion, which could not effectively prove that the particles formed a network structure in the matrix. Therefore, the motion process of PVP@PANI particles in the PDMS matrix under the action of an external electric field was observed in real time by an optical microscope. By observing the movement process of PVP@PANI particles in the PDMS matrix under the action of an external electric field in real time by optical microscopy, the mechanism of the particles forming a network structure under the action of an external electric field could be understood in detail. The specific explanation and process are as follows: Before applying the electric field, that is, at t = 0 s, it can be observed that the particles are randomly dispersed in the PDMS matrix. After starting the application of the electric field with an electric field strength E = 1000 V p-p/mm and frequency f = 10 Hz, the PVP@PANI particles quickly moved and aligned along the direction of the electric field to form a network structure. As the orientation time increased, that is, when t = 50 s, the formed network structure was more complete and compact.

**Figure 5:**
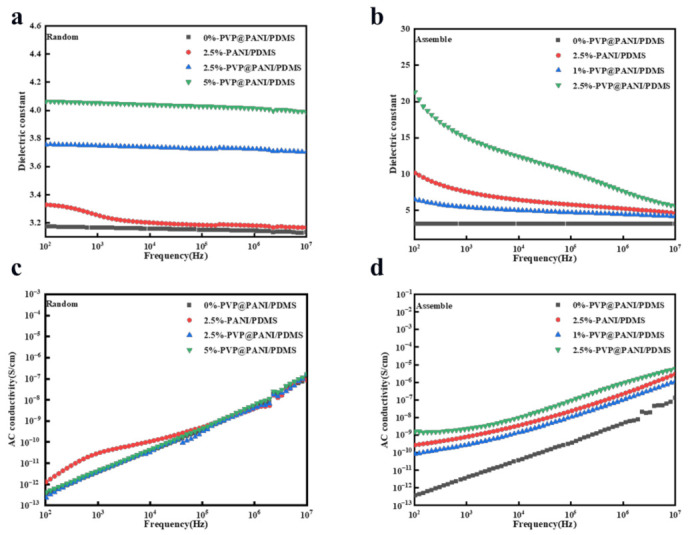
Dielectric constant and electrical conductivity of PVP@PANI/PDMS composites and PANI/PDMS composites with different PVP@PANI contents. (**a**) Dielectric constant of random composite. (**b**) Dielectric constant of assembled composite. (**c**) Conductivity of random composite. (**d**) Conductivity of assembled composite.

We systematically investigated the effects of network structure and filler content on the dielectric properties of composites. The dielectric permittivity and dielectric loss tangent of the composite are shown in Figure 5. The dielectric permittivity of the assembled composite films (cured with electric fields) was apparently higher than random ones (cured without electric fields). For example, for 2.5 wt% PVP@PANI composites, the dielectric permittivity of the assembled composite films could reach up to 22.5 at 10^2^ Hz, which is nearly 6 times higher than randomly dispersed composites (control samples). The dielectric constant of the assembled PVP@PANI composite film (electric field curing) was also significantly higher than that of the PANI composite film (electric field curing). For example, for the 2.5 wt% PANI composite, the dielectric constant of the assembled composite film was 10.1 at 10^2^ Hz. The dielectric permittivity of both random and assembled PVP@PANI/PDMS composites increased with the increase in PVP@PANI loading. The increase in the dielectric constant is attributed to the formation of micro-capacitors and the enhancement of interfacial polarization in the PVP@PANI/PDMS composite films, which usually occurs in dielectric composites with conductive fillers [21,23,32]. PANI is a conductor, and a conductive path or network can be formed in the matrix, which greatly improves the ability of the composite material to store electric charge. PVP is a highly polar polymer with excellent electrical insulation [33]. The pyrrolidone ring in the PVP structure contains a double-bonded carbonyl group, which is attached to the main chain through an amide bond. This lactam active group of PVP will be in a reactive state at a certain temperature, and can form charge transfer complexes with many substances. This group also enables it to have good charge storage performance [34]. However, the ability of the assembled PVP@PANI composites to store charge increases due to the formation of networks in the PDMS matrix, and thus the dielectric constant is greatly improved [35]. The decrease in dielectric permittivity of composites with increasing frequency can be explained by the Maxwell−Wagner−Sillars polarization, because the polarization cannot catch up with the change in direction of the external electric field at higher frequencies. Therefore, the particles and interfaces in the composite can only be fully polarized at low frequencies, leading to higher dielectric constants [36,37,38]. Figure 5c,d shows the effect of frequency on the conductivity of PVP@PANI/PDMS films. The conductivity of all samples increased slightly with increasing PVP@PANI particle content, but also exhibited strong frequency dependence over the entire frequency range. Pure PDMS was completely insulating with a conductivity of 3.5 × 10^−13^ at 10^2^ Hz; and a composite film with a PVP@PANI particle content of 2.5 wt% had a conductivity of 1.5 × 10^−9^. All the composite films are considered as insulating materials, which indicates that adding conductive PANI will not form a conductive path. [39]. Table 1 summarizes some reported PDMS composite dielectric materials and PVP@PANI/PDMS dielectric constants prepared using an electric field. The comparison shows that PVP@PANI/PDMS exhibited a higher dielectric constant with a small amount of addition.

**Figure 6:**
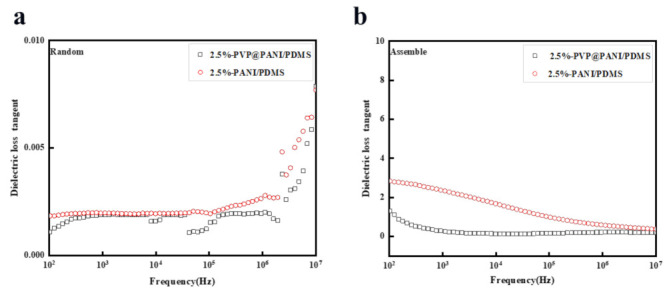
Dielectric loss tangent of (**a**) random PVP@PANI/PDMS and PANI/PDMS composites, (**b**) assembled composites.

As shown in Figure 6a,b, all PVP@PANI/PDMS composite films exhibited relatively low dielectric loss tangents in a wide frequency range (10^2^ Hz to 10^7^ Hz). It is worth noting that the dielectric constant of the composite membrane material prepared under the electric field increases while keeping the dielectric loss tangent at a very low value. For samples containing PVP@PANI, the dielectric loss tangent is only 1 when adding 2.5 wt% PVP@PANI, lower than that of aligned samples PANI. This is because the presence of PVP between conductive PANI in aligned channels can block the formation of conductive paths and minimize the increase in dielectric loss tangent caused by the leakage of current. The insulated PVP shell reduces the dielectric loss tangent, which acts as a dielectric layer to suppress the current leakage between PANI in direct contact [40,41,42,43].

## 4. Conclusions

In conclusion, we have developed a new strategy for facilitating particles in forming a network structure within a matrix under an applied electric field, and fabricated high dielectric constant polymer composites. By incorporating PVP@PANI particles into PDMS and forming a network through an electric field, polymer composites with high dielectric constant and low dielectric loss tangent can be obtained. The dielectric constant can be significantly increased to 23 after adding 2.5 wt% PVP@PANI.

## Figures and Tables

**Table 1:** Reported properties of similar dielectric composites and our PVP@PANI/PDMS (where PDMS is polydimethylsiloxane) prepared by electric field [24,30].

Polymer Composition	Filler Particle Mass Fraction (wt%)	Dielectric Constant at 10^2^ Hz	Dielectric Constant at 10^3^ Hz	Dielectric Loss Tangent at 10^3^ Hz
Pure PDMS	-	3.2	3.19	0.0125
CCTO/PDMS	1	3.7	3.5	0.02
PANI@CCTO/PDMS	1	4.6	4.25	0.025
PVP@BT/PDMS	10	3.5	3.45	0.05
PVP@BT/PDMS	20	3.7	3.7	0.06
PVP@PANI/PDMS	1	7	5	0.08
PVP@PANI/PDMS	2.5	23	15	0.23

## Data Availability

The data that support the findings of this study are available from the corresponding author, Yuwei Chen, upon reasonable request.
